# Multicenter Testing of a Simple Molecular Diagnostic System for the Diagnosis of Mycobacterium Tuberculosis

**DOI:** 10.3390/bios13020259

**Published:** 2023-02-12

**Authors:** Hyo Joo Lee, Nam Hun Kim, Eun Hye Lee, Young Soon Yoon, Yun Jeong Jeong, Byung Chul Lee, Bonhan Koo, Yoon Ok Jang, Sung-Han Kim, Young Ae Kang, Sei Won Lee, Yong Shin

**Affiliations:** 1 Department of Biotechnology, College of Life Science and Biotechnology, Yonsei University, 50 Yonsei-ro, Seodaemun-gu, Seoul 03722, Republic of Korea; 2 INFUSIONTECH, 38 Heungan-daero, 427 Beon-gil, Dongan-gu, Anyang-si 14059, Republic of Korea; 3 Division of Pulmonology, Allergy and Critical Care Medicine, Department of Internal Medicine, Yongin Severance Hospital, Yonsei University College of Medicine, Yongin-si 06273, Republic of Korea; 4 Division of Pulmonology and Critical Care Medicine, Department of Internal Medicine, Dongguk University Ilsan Hospital, Dongguk University College of Medicine, Goyang-si 10326, Republic of Korea; 5 Department of Infectious Diseases, Asan Medical Center, University of Ulsan College of Medicine, Songpa-gu, Seoul 05505, Republic of Korea; 6 Division of Pulmonary and Critical Care Medicine, Department of Internal Medicine, Severance Hospital, Yonsei University College of Medicine, Seoul 03722, Republic of Korea; 7 Department of Pulmonary and Critical Care Medicine, Asan Medical Center, University of Ulsan College of Medicine, Seoul 05505, Republic of Korea

**Keywords:** tuberculosis, molecular diagnostics, sample preparation, NAs extraction

## Abstract

*Mycobacterium tuberculosis* (MTB) is a communicable disease and still remains a threat to common health. Thus, early diagnosis and treatment are required to prevent the spread of infection. Despite the recent advances in molecular diagnostic systems, the commonly used MTB diagnostic tools are laboratory-based assays, such as mycobacterial culture, MTB PCR, and Xpert MTB/RIF. To address this limitation, point-of-care testing (POCT)-based molecular diagnostic technologies capable of sensitive and accurate detection even in environments with limited sources are needed. In this study, we propose simple tuberculosis (TB) molecular diagnostic assay by combining sample preparation and DNA-detection steps. The sample preparation is performed using a syringe filter with amine-functionalized diatomaceous earth and homobifunctional imidoester. Subsequently, the target DNA is detected by quantitative PCR (polymerase chain reaction). The results can be obtained within 2 h from samples with large volumes, without any additional instruments. The limit of detection of this system is 10 times higher than those of conventional PCR assays. We validated the clinical utility of the proposed method in 88 sputum samples obtained from four hospitals in the Republic of Korea. Overall, the sensitivity of this system was superior to those of other assays. Therefore, the proposed system can be useful for MTB diagnosis in limited-resource settings.

## 1. Introduction

Tuberculosis (TB) is a communicable disease caused by *Mycobacterium tuberculosis* (MTB), which can spread through the air, for example, by coughing. TB is a major cause of health deterioration and death worldwide [1]. TB affects multiple organs; it can be classified as a multisystem infectious disease. The infection rate is so high that about a quarter of the world’s total population is estimated to be infected with tuberculosis [2,3]. In 2015, the United Nations established 17 Sustainable Development Goals, which include ending tuberculosis by 2030. However, tuberculosis research is relatively obscured by the allocation of resources such as manpower, laboratories, and clinical services for diseases such as HIV, malaria, and now, COVID-19 [4,5,6,7].

TB, like other communicable diseases, requires rapid diagnosis and treatment in the early stages. Tuberculosis diagnostic tests have improved significantly in recent years and may involve chest X-ray, tuberculin test, microscopic observations of sputum smears, bronchoscopy, CT (computed tomography) scan, sputum culture, and tissue biopsy analysis. Among these, acid-fast bacilli (AFB) smear microscopy and bacterial culture are used as the gold standards [1,8]. However, the sensitivity of pulmonary tuberculosis identification through microscopic smear tests is estimated to be up to 70% [9]. The mycobacterial culture method requires 2–8 weeks for cultivating the bacteria, and there is a limit to the minimum number of bacteria that can be detected [8,10]. The analysis of sputum after acid-fast (AF) staining is faster and easier for the diagnosis of pulmonary TB compared with sputum culture. AF staining is cost-effective, relatively simple, and fast. Nevertheless, sample processing, the thickness of the smears, the preparation and conservation of the reagents, the quality of the microscopes, the duration of the primary and counterstaining incubations, as well as the expertise of the technical staff affect the sensitivity and specificity of AF staining [11]. Additional studies have been conducted to achieve better efficiency using the existing methods. For instance, digital chest x-rays with the computer-aided detection of tuberculosis have been increasingly used in various settings. However, this technique still needs improvements in computer-aided detection [12].

Currently, several molecular diagnostic technologies are available for the detection of TB with high sensitivity and specificity. Nucleic acid amplification tests (NAATs) particularly emerged as an alternative to traditional methods as they are faster and easier to apply [13,14]. Several fast molecular tests are recommended by the WHO as initial diagnostic tests for TB, such as Xpert MTB/RIF Ultra (Ultra) (Cepheid, Sunnyvale, CA, USA) and Truenat MTB/RIF (Molbio Diagnostics, Verna, India), some of which can simultaneously detect drug resistance [1,15]. However, there are still many regions in the world that rely heavily on outdated tuberculosis diagnostic tests [16,17,18]. These methods are still used despite their poor sensitivity as the cost and infrastructure requirements of molecular testing methods do not allow scaling up, and the available resources of molecular testing are often underutilized [19,20]. To address the limitations of conventional diagnostic technologies, point-of-care testing (POCT)-based technologies have been researched. POCT performs well as a simple, rapid, low-cost method even in resource-limited environments. POCT has been extensively studied in relation to biosensors [21], microfluidic systems [8,22], and lipoarabinomannan (LAM) tests [23].

In this study, we report a new molecular diagnostic system for TB detection. This system was developed by integrating a simple sample preparation technology with a DNA detection technology. For sample preparation, we used a simple DNA extraction method comprising a syringe and a syringe filter with homobifunctional imidoesters (HIs), like dimethyl suberimidate (DMS), that bind to amine-functionalized diatomaceous earth and amine groups [24]. This sample preparation system enables DNA extraction in about 1–2 h depending on the sample volume (up to 10 mL) with inexpensive and robust. This technology has been proven effective as it involves simple procedures and does not require any instruments. Then, the extracted DNA was analyzed by quantitative PCR using specific primers. Additionally, we validated the clinical utility of the system in 88 sputum samples collected from patients. Therefore, this system provides a diagnosis method that is more rapid and simple than the traditional TB diagnostic assays, such as Xpert MTB/RIF, MTB PCR, and mycobacterial culture.

## 2. Materials and Methods

### 2.1. Chemicals and Reagents

Hyflo Super Cel (Diatomaceous earth), 3-aminopropyl(diethoxy)methylsilane (APDMS, 97%), dimethyl suberimidate dihydrochloride (DMS, 98%), lysozyme solution (50 mg/mL in distilled water), sodium hydroxide solution (50% in H_2_O), N-Acetyl-L-cysteine (NALC, 99%), sodium citrate, and Triton X-100 were purchased from Sigma-Aldrich (St. Louis, MO, USA). Tris-HCI (pH 8.0), distilled water (DNase/RNase-Free), and EDTA (pH 8.0) were purchased from Invitrogen (Waltham, MA, USA). Proteinase K solution (>600 mAU/mL) was purchased from Qiagen (Hilden, Germany). Absolute ethanol was purchased from Merck (Whitehouse Station, NJ, USA). Phosphate-buffered saline (PBS; 10×, pH 7.4) was purchased from Gibco (Grand Island, NY, USA).

### 2.2. Synthesis of Amine-Functionalized Diatomaceous Earth(D-APDMS)

D-APDMS used in the NA extraction processes was prepared as follows [24,25,26]. Diatomaceous earth (DE) was washed with distilled water (DW) for 30 min with stirring. The sediment containing impurities was removed after a short period of settling under gravity. APDMS was used to prepare D-APDMS. Briefly, 5 mL of APDMS was pipetted dropwise into 100 mL 95% (*v*/*v*) ethanol solution, which was acidified with acetic acid (pH 5) and combined with 2 g DE. The mixture was incubated for 4 h at room temperature (RT) with stirring. Then, D-APDMS was washed with ethanol, dried under vacuum overnight, and stored at RT until use.

### 2.3. Filter-Based Nucleic Acid Extraction

D-APDMS and DMS were used as the matrix for bacterial DNA extraction. First, 30 μL of lysozyme was mixed with 1.5 mL of sample, and the mixture was incubated for 1 h at 37 °C. After incubation, 1 mL of D-APDMS suspension (60 mg/mL in 10 mM Tris-HCl buffer at pH 7.0), 1 mL of DMS solution (100 mg/mL in 70% ethanol), 1 mL of GTIC lysis solution (4 M GITC, 55 mM Tris-HCI, 25 mM EDTA, 3% Triton X-100 in distilled water), and 50 μL of Proteinase K were pipetted into the sample solution. Then, the mixture was incubated for 30 min at 56 °C and 15 min at 95 °C for NA extraction. During the incubation, a hydrophobic PTFE syringe filter (25 mm, 3.0 μm, Hawach Scientific, Xi’an, China) was washed with 1 mL PBS. The incubated mixture was transferred into a syringe filter and then washed with 2 mL of PBS using the syringe. Finally, 150 μL of elution buffer (10 mM Tris-HCl, pH 10.0) was added into the syringe filter and, after 1 min of incubation at RT, the elution buffer containing NAs was collected, and the extracted DNA was stored at −20 °C until use.

### 2.4. Nucleic Acid Detection Method

Isolated DNA was analyzed by quantitative PCR to examine the efficiency of the sample preparation process using D-APDMS. Quantitative PCR conditions were as follows: an initial denaturation step at 95 °C for 15 min; 45 cycles of incubation at 95 °C for 10 s, at 63 °C for 20 s, and at 72 °C for 20 s; and melting steps at 95 °C for 30 s, at 65 °C for 30 s, and at 95 °C for 30 s. Amplification was performed in a total volume of 20 µL reaction mixture containing 5 µL of DNA, 10 µL of AccuPower 2× GreenStar qPCR Master Mix (Bioneer, Daejeon, Republic of Korea), and 2.5 µM of each primer. We performed conventional PCR, quantitative PCR, and recombinase polymerase amplification (RPA) to determine the quality of DNA extracted from TB. The conventional PCR cycling conditions were as follows: an initial denaturation step at 95 °C for 15 min; 40 cycles of incubation at 95 °C for 30 s, 58 °C for 30 s, and 72 °C for 30 s; and a final extension step at 72 °C for 5 min. The mixture included 5 µL of DNA in a total volume of 25 μL containing PCR buffer (10×, Qiagen), 2.5 mM MgCl_2_, 0.25 mM deoxynucleotide triphosphate, 25 pM of each primer, one unit of Taq DNA polymerase (Qiagen), and deionized (DI) water. The quantitative PCR conditions were as follows: an initial denaturation step at 95 °C for 30 s; 40 cycles of incubation at 95 °C for 5 s, 60 °C for 30 s; and cooling at 40 °C for 30 s. Amplification reactions contained 5 μL of RNA and were performed with LightCycler^®^ Multiplex RNA Virus Master (Roche, Mannheim, Germany). PCR products were analyzed by electrophoresis on a 2% agarose gel. The gel was visualized using a ChemiDoc XRS+ System (Bio-Rad, Hercules, CA, USA). The RPA reaction was performed using 3 μL of RNA and a TwistAmp^®^ RT Basic kit (TwistDx, Cambridge, UK) for 25 min at 40 °C. RPA products were analyzed on a 2% agarose gel and by lateral flow assay (LFA) using a Milenia HybriDetect 1 kit (TwistDx). Clinical samples of TB were confirmed using the PrimeraTM TB/MDR-TB Detection Kit (Cat Nr. PRT021 Infusion Tech, Gyeonggi-do, the Republic of Korea) (Figure S1, Table S2) according to the manufacturer’s instructions. Conventional PCR and RPA were performed on a T100 Thermal Cycler (Bio-Rad, Hercules, CA, USA). All quantitative PCR assays were performed on a CFX96 Touch Real-Time PCR Detection System (Bio-Rad).

### 2.5. Bacteria Samples and Clinical Samples

**Figure 1:**
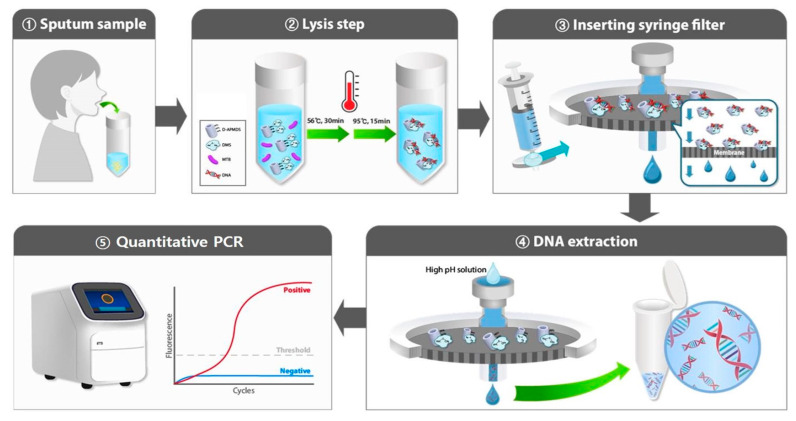
Schematic representation of the principle of the proposed molecular diagnosis system. This system consists of a sample preparation step and a DNA detection step. (**1**) Collect a sputum sample from the patient. (**2**) In the lysis step, added D-APDMS and DMS to the sample. During incubation, Bacteria are lysed out and DNA comes out, and DNA from bacteria binds to D-APDMS with DMS as a cross-linker. (**3**) Insert the mixture into a syringe filter to separate the D-APDMS-captured DNA. (**4**) By adding a high-pH solution, the binding of D-APDMS with DNA is broken. Then, the DNA is collected in the tube. (**5**) Finally, the extracted DNA is analyzed through quantitative PCR.

To investigate the capacity of D-APDMS with syringe filter assays for bacterial cells, we used the extracted DNA from *Brucella ovis* (ATCC 25840) cells, which were grown in Brucella agar containing 5% defibrinated sheep blood and incubated at 37 °C in (5% CO_2_) for 48 h. All patients with suspected pulmonary TB (PTB) who consented to the use of their sputum for additional tests, such as the TB diagnostic platform (Figure 1), were prospectively enrolled at a 2700-bed tertiary-care facility in Republic of Korea (Severance Hospital and Asan Medical Center, Dongguk University Ilsan Hospital, Yongin Severance Hospital, IRB 4-2020-1177. 2018-0020, 2020-1745, 2021-03-032-003, 9-2020-0166), and the protocol of this study was registered at clinicaltrials.gov (NCT03423550). The suspicion of PTB was based on the participants’ symptoms, history, and radiographic findings suggestive of TB. The enrollment was decided by five respiratory and infection specialists (E.H.L., Y.S.Y., S.H.K., Y.A.K., and S.W.L.), who had more than 15 years of experience in TB treatment. One volume of liquefaction buffer (5% NaOH, 4% NALC, and 1.5% sodium citrate in distilled water) was added to the collected clinic sputum sample. Then, it was stored at −20 °C until use.

## 3. Results and Discussion

### 3.1. Principles of TB Molecular Diagnostic System

The process of the tuberculosis molecular diagnostic system proceeds in five steps comprising sample preparation and detection stages (Figure 1). First, sputum samples are collected from the patients (Figure 1(1)) and mixed with the liquefaction buffer to obtain a liquid sample. Then, lysozyme is added to disrupt the cell membrane, and the samples are incubated at 37 °C for 60 min. Samples in large volumes (up to 10 mL) can be used for TB diagnosis without requiring additional instruments. Second, in the bacteria lysis step (Figure 1(2)), the lysis buffer is added with D-APDMS and DMS, and the samples are incubated for 30 min at 56 °C and 15 min at 95 °C for bacteria lysis. During the lysis step, DMS is added to bind between DNA and D-APDMS. DMS has imido groups on both sides, which bind to the amine groups on the surface of D-APDMS on one side and the amine groups on DNA on the other side by covalent bonding. Since DNA has a negative charge, it also participates in electrostatic interactions. These interactions are stably maintained at pH 8. Third, a PTFE syringe filter is used to separate the DNA-bound D-APDMS (Figure 1(3)). The size of DE ranges from 200 nm to 3 μm, which is bigger than the filter membrane. Therefore, the D-APDMS is collected in the membrane, and other unnecessary substances are removed by washing with PBS. Fourth, the elution buffer is used to break the interactions. The extracted DNA is simply collected (Figure 1(4)) and confirmed by quantitative PCR (Figure 1(5)). This TB molecular diagnostic system enables nucleic acid extraction and detection within 3 h without requiring chaotropic agents or sophisticated instruments.

### 3.2. Optimization and Application of D-APDMS with Syringe Filter

**Figure 2:**
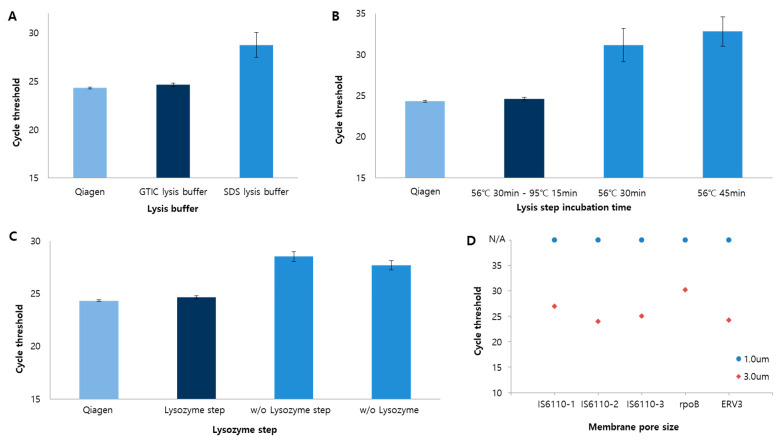
Optimization of the D-APDMS with syringe filter platform. (**A**) The efficiency of sample lysis depends on the lysis buffer. The results showed that the GITC lysis buffer (dark blue) was more efficient than the sodium dodecyl sulfide (SDS) lysis buffer. (**B**) Cell lysis is affected by incubation time and temperature. Upon testing under three conditions, the best results were obtained with incubations at 56 °C for 30 min and at 95 °C for 15 min (dark blue). (**C**) The efficiency of the lysozyme. Higher efficiency was obtained with the additional lysozyme step (dark blue). Data are presented as the mean ± SD based on at least three independent experiments. (**D**) The effect of pore size on DNA extraction efficiency.

Prior to the use of the TB molecular diagnostic system, an optimization experiment for the use of D-APDMS with a syringe filter was performed. The performance of D-APDMS in sample preparation was confirmed through experiments using amine-functionalized diatomaceous earth [24,25,26]. All optimization experiments were performed with the same concentration of *Brucella ovis* 10^5^ CFU/mL, and the efficiency of the system was compared with that of a commercial column kit. Several conditions were tested in the lysis steps. SDS-based lysis buffer and GITC-based lysis buffer were compared (Figure 2A), and the GITC-based lysis buffer was found to be more effective as indicated by lower Ct values. Then, the incubation duration of the lysis step was optimized (Figure 2B), and it was observed that a 30 min incubation (56 °C) produced better results than a 45 min incubation. To further increase the extraction efficiency, we tried combining the thermal lysis method with the existing chemical lysis method. We compared the efficiencies of the following lysis procedures by examining the Ct values: (i) 30 min incubation at 56 °C followed by an additional 15 min at 95 °C and (ii) a single step of 30 min incubation at 56 °C. The former, i.e., the extra 15 min incubation at 95 °C after the 30 min incubation at 56 °C resulted in superior efficiency. Next, the optimization of the lysozyme, which breaks the bacterial cell membrane, was also performed (Figure 2C). Since the optimum temperature for lysozyme activity was 37 °C, the lysozyme step (1 h at 37 °C) was added before the lysis step. Compared to the addition of lysozyme before or during lysis, the use of lysozyme before the lysis step resulted in better efficiency (Figure 2C). Taken together, it was confirmed the better efficiency of the system to enrich and extract DNA at 56 °C for 30 min and 95 °C for 15 min in a GITC-based lysis buffer. In addition, a lysozyme step was added before the lysis step to increase the efficiency of the cell lysis step. Under these conditions, bacteria enrichment and the DNA extraction process were conducted using a syringe filter and D-APDMS was determined.

Next, we found that the syringe filter was blocked by various contaminants during the addition of sputum samples. We performed DNA extraction using filter membranes with a pore size of either 1.0 μm or 3.0 μm (Figure 2D). The extracted DNA was analyzed by quantitative PCR. *ERV3* gene was used as an internal control; *IS6110* and *rpoB* genes were selected as TB targets. No amplification was observed in quantitative PCR in samples prepared using the 1.0 μm filter, presumably because the filter was blocked. On the other hand, efficient amplification with the expected Ct values was obtained with the 3.0 μm filter. Consequently, it was determined to conduct the experiments on the clinical samples using 3.0 μm filters.

**Figure 3:**
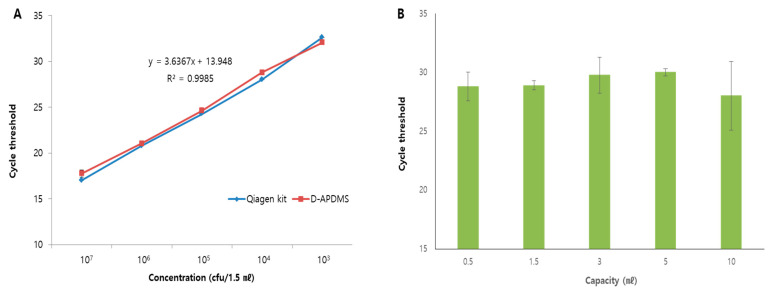
Application of the D-APDMS with syringe filter platform. (**A**) The efficiency of DNA extraction was confirmed at various concentrations. The test was performed for a concentration range from 1 × 10^7^ to 1 × 10^3^ CFU/1.5 mL using a Qiagen kit (blue) and the D-APDMS platform (red). (**B**) DNA extraction was tested using samples with volumes ranging from 0.5 to 10 mL. The data are presented as the mean ± SD based on at least three independent experiments.

Next, we tested the extraction capacity of the system using various concentrations of bacteria ranging from 10^3^ CFU to 10^7^ CFU per 1.5 mL (Figure 3A). DNA was successfully extracted from samples containing both low and high concentrations of bacteria (Figure 3A), and no difference in extraction efficiency was found between the proposed system and a commercial spin column kit. In addition, we also performed tests with the same concentration (Brucella ovis 10^5^ CFU/mL) of bacteria at different volumes ranging from 0.5 to 10 mL. DNA extraction was possible from both small and large volumes of samples (up to 10 mL; Figure 3B), suggesting that our method might overcome the limitations of commercial kits that are applicable to limited volumes of samples. Consistent with previous studies, the limit of detection can be enhanced when a large volume of sample used during sample preparation [27,28,29]. This proposed system can be enriched and extracted DNA from a large volume of samples. Based on this advantage of this system, it can show better clinical sensitivity for the detection of MTB.

### 3.3. Testing of Various DNA Amplification Methods for TB Molecular Diagnostics

**Figure 4:**
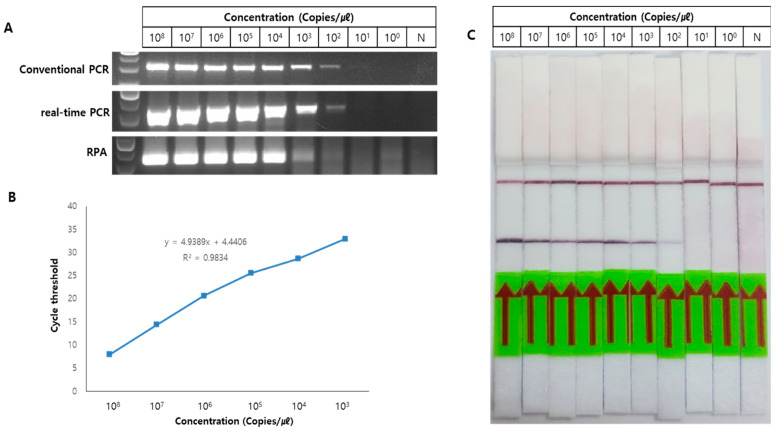
TB detection using various amplification methods. (**A**) The sensitivities of indicated amplification methods. The reaction products could be detected (1 × 10^8^ to 1 copies of DNA) on a 2% agarose gel. Conventional PCR (**up**) and quantitative PCR (middle) detected DNA amounts as low as 10^2^ copies. Detection limit of RPA (**down**) was 10^3^. (**B**) A standard curve of quantitative PCR data. This result indicates that quantification is possible. (**C**) LFA was performed using the product amplified by RPA, resulting in a detection limit of 10^3^.

To determine the optimal method for TB molecular diagnostics, we tested the detection limits of various amplification methods including conventional PCR, quantitative PCR, RPA, and paper-based lateral flow assay (LFA). We used ten-fold serially diluted DNA samples ranging from 1 to 10^8^ copies. The products of conventional PCR, quantitative PCR, and RPA were analyzed on a 2% agarose gel. We found that the detection limits of conventional PCR and quantitative PCR were the same (10^2^ copies; Figure 4A). The gene *IS6110* was used as a marker of TB, and the gene *ERV3* was used as an internal control. Based on this, we drew a standard curve (Figure S1, Table S2). When the detection limit was calculated for a Ct value of 38, which is the standard set by the company, the resultant standard curve indicated a detection limit of about nine copies for the two genes. On the contrary, RPA has the advantages of desirable isothermal properties and speed, but it has a higher detection limit (10^3^ copies). These results are likely to be sufficient for in-field diagnosis using RPA, but the efficiency was slightly reduced (Figure 4B). Additionally, for the possibility of an in-field diagnosis of TB, LFA was performed using the RPA product (Figure 4C), which exhibited a detection limit similar to that of RPA. Compared with testing of various detection methods with the D-APDMS platform, RPA and LFA methods are still required for improvement of the efficiency. Based on the test results of the detection methods, we decided to proceed with conventional PCR and quantitative PCR, which showed the best efficiency. Although quantitative PCR has the same detection limit as conventional PCR, it does not require additional steps, such as 2% agarose gel electrophoresis, and takes about 1 h, which is shorter than conventional PCR (>2 h). Therefore, quantitative PCR was selected as the detection method for the TB molecular diagnostic system.

### 3.4. Utility of the TB Molecular Diagnostic System on Clinical Samples

**Figure 5:**
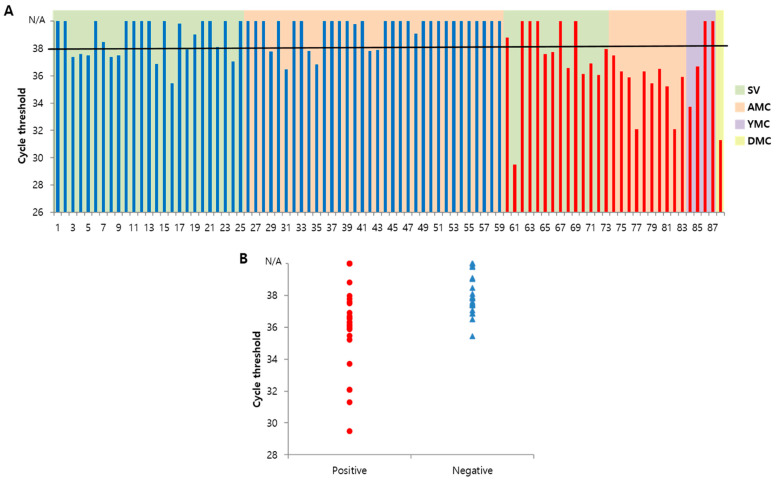
Application of TB diagnosis in clinical samples using the simple molecular diagnostic system. (**A**) Results obtained using a simple molecular diagnostic system in 88 clinical samples. Samples from TB-positive patients are marked in red and those from TB-negative patients in blue. The collected center marked differences in the background color; light green Severance Hospital (SV) and light orange Asian Medical Center (AMC), light purple Yongin Severance Hospital (YMC), and light yellow Dongguk University Ilsan Hospital (DMC). The diagnostic result of this system was based on the Ct threshold of 38. (**B**) The distribution of Ct values by positive (red) and negative (blue) samples. In positive samples, the number of samples with below a Ct value of 38 was more distributed, in negative samples, the number of samples with above a Ct value of 38 was more distributed.

To validate the clinical utility of the system, we examined 88 sputum samples obtained from patients at four hospitals in the Republic of Korea (Severance Hospital and Asan Medical Center, Dongguk University Ilsan Hospital, Yongin Severance Hospital). The sputum samples were analyzed through the TB molecular diagnostic system consisting of a DNA extraction (D-APDMS with syringe filter) step followed by a DNA detection (quantitative PCR) step. Figure 5A shows the Ct values obtained. The test result was considered to be negative for Ct values of 38 or higher and positive for Ct values below 38. All samples were tested using the TB molecular diagnostic system, Xpert MTB/RIF, MTB PCR, AFB smear, and mycobacterial culture assays (Table 1). The clinical samples consisted of 29 TB-positive and 59 TB-negative samples, as determined by results of mycobacterial culture (culture positive and culture negative). TB molecular diagnostic system detected 21 out of 29 samples as true positives and 44 out of 59 samples as true negatives. Thus, the sensitivity and specificity of the assay were 72.41% and 74.58%, respectively. The sensitivities of Xpert MTB/RIF, MTB PCR, and AFB smear were 50%, 27.27%, and 13.79%, respectively. Nevertheless, the specificity was 100% for all TB assays. Of note, the sensitivity of the proposed system was about 300–500% higher than those of the MTB PCR and AFB smear assays. Overall, the data indicated that the sensitivity of the TB molecular diagnostic system was superior to those of conventional TB diagnosis assays.

## 4. Conclusions

There are several assays available for disease diagnosis. POCT-based diagnosis methods, which are not laboratory-based assays, have been developed. TB diagnosis assays need to be simplified to provide POCT in a real setting. In this work, we report a simplified molecular diagnostic system for TB detection. This system allows rapid and simpler diagnosis of TB compared with the traditional assays. In particular, the sample preparation step was shortened and simplified, resulting in a potential POCT system.

The proposed system has several advantages. First, the proposed sample preparation method enables DNA extraction in about 1–2 h depending on the sample volume (up to 10 mL). High amounts of DNA can be extracted from large samples without reducing detection efficiency. This sample preparation system uses non-chaotropic reagents for capturing MTB and extracting DNA without requiring any specific instrument. This system also helps to increase sensitivity to detection by conducting bacteria enrichment and DNA extract using syringes and syringe filters to simplify the sample preparation step. Second, the utility of DNA extraction was confirmed in clinical samples, proving through real-time PCR is a possibility to be applied to clinical on-site. Third, the membrane of MTB is too thick to be disrupted using common lysis buffers. We optimized the lysis buffer using lysozyme to efficiently break the cell membrane. Fourth, compared to AFB smear or Mycobacterial culture assay (takes 2–8 weeks) [30,31], this system is a low-cost and rapid-based assay in a limited environment by a simple protocol (Supplementary Table S4). It has a high potential to be applied as a POCT method in the future. Fifth, we examined various assays for TB diagnosis. Among those, quantitative PCR was selected as the optimal detection method for TB using specific primers. Although the use of quantitative PCR may not be suitable for POCT, the sensitivity of the whole system including the sample preparation step was three times superior to that of MTB PCR assay. Table 1 shows the comparison of the sensitivity and specificity indices of the proposed system and other assays presented in this study.

Based on these results, further research is needed to overcome the limitations of this study. First, the two techniques, sample preparation and detection, need to be integrated for POC testing with automation. After integration, the operation steps for the system should be minimized. Several hands-on steps are still required for the reaction. Second, a novel detection technique should be investigated for POC testing to replace quantitative PCR. Because quantitative PCR has several limitations; it requires large instruments, takes time, and is expensive, highly limiting its use for POC testing. Third, although the sensitivity of this system was superior to other assays, the specificity should be improved. It is because of the false-positive results in the clinical samples. The reasons for the false-positive results were not controlled. It may be related to the high sensitivity of this system due to the detection of remnant bacilli or DNA from previous infections. Further study is needed to overcome the limitations. Fourth, this was a proof-of-concept study for the system for TB detection. Although the clinical utility of the system was demonstrated by testing 88 clinical samples, this work is not sufficient to conclusively evaluate the performance of the system. Therefore, further studies would be needed for a better examination of the performance of the system with a larger cohort of clinical samples. Fifth, other types of liquid biopsies should be analyzed using this system to examine the versatility of the system. Finally, recent advances in nanotechnology might be investigated for their implications for TB molecular testing in a real setting [32]. Nevertheless, this TB molecular diagnostic system can be useful for TB diagnosis as it provides a rapid and simple assay with high sensitivity and specificity. Therefore, we envision that this system, which combines sample preparation and detection, enables simple and rapid diagnosis of disease with clinical applications for communicable and non-communicable diseases.

## Figures and Tables

**Table 1:** Comparison of specificity and sensitivity in clinical samples.

	This Study	Xpert MTB/RIF	MTB PCR	AFB Smear	Mycobacterial Culture
Sensitivity (95% CI)	72.41% (52.76–87.27%)	50% (30.65–69.35%)	27.27% (6.02–60.97%)	13.79% (3.89–31.66%)	100% (88.06–100%)
Specificity (95% CI)	74.58% (61.56–85.02%)	100% (93.84–100%)	100% (76.84–100%)	100% (93.94–100%)	100% (93.94–100%)

## Data Availability

The data that support the findings of this study are available from the corresponding author upon reasonable request.

## Supplementary Materials

The following supporting information can be downloaded at: https://www.mdpi.com/article/10.3390/bios13020259/s1, Figure S1: The efficiency of the TB detection kit; Table S1: Sequences of primer sets; Table S2: Results of the TB detection kit for standard curve; Table S3: Results of the experiment for 88 clinical samples; Table S4: Comparison of the new molecular diagnostic system with other TB detection methods.
