# Supplementary material for: Multicenter Testing of a Simple Molecular Diagnostic System for the Diagnosis of Mycobacterium Tuberculosis
Source: Biosensors (Basel). 2023 Feb 12;13(2):259. doi: 10.3390/bios13020259 (PMC9954000; doi:10.3390/bios13020259)
Supplement: Supplementary file 1 [file biosensors-13-00259-s001.zip › biosensors-2159664-supplementary.pdf]

biosensors

Supplementary Material

Multicenter Testing of a Simple Molecular Diagnostic System for the Diagnosis of Mycobacterium Tuberculosis

Hyo Joo Lee [1,†], Nam Hun Kim [2,†], Eun Hye Lee [3], Young Soon Yoon [4], Yun Jeong Jeong [4], Byung Chul Lee [2], Bonhan Koo [1], Yoon Ok Jang [1], Sung-Han Kim [5], Young Ae Kang [6,*], Sei Won Lee [7,*] and Yong Shin [1,*]

Abbreviations.

MTB	*Mycobacterium tuberculosis*
TB	Tuberculosis
PTB	Pulmonary Tuberculosis
AFB	Acid-fast bacilli
NAATs	Nucleic acid amplification tests
POCT	Point-Of-Care test
HI	Homobifunctional Imidoester
DMS	Dimethyl suberimidate
APDMS	3-aminopropyl(diethoxy)methylsilane
DE	Diatomaceous earth
D-APDMS	Amine-Functionalized Diatomaceous Earth
NA	Nucleic Acid
SD	standard deviation
DW	Distilled Water
PBS	Phosphate-buffered saline
PCR	Polymerase Chain Reaction
RPA	Recombinase-based polymerase amplification
LFA	lateral flow assay

Table S1. Sequences of primer sets.

Primer	Sequences (5′–3′)
Brucella-F	GCTTGAAGCTTGCGGACAGT
Brucella-R	GGCCTACCGCTGCGAAT
TB_IS6110_PCR-F	TTAAAGACCGCGTCGGCTTTC
TB_IS6110_PCR-R	ACGGTTCAGGGTTAGCCACA
TB_IS6110_RPA-F	FAM-TTAAAGACCGCGTCGGCTTTCTTCGCGGCCGA
TB_IS6110_RPA-R	Biotin-ACGGTTCAGGGTTAGCCACACTTTGCGGGCAC
TB_IS6110_1_F	GACCTACTACGACCACATCAAC
TB_IS6110_1_P	6FAM-ATGGCGAACTCAAGGAGCACATCA-BHQ-1
TB_IS6110_1_R	GTTCAGGGTTAGCCACACTT
TB_IS6110_2_F	GGACCACCAGCACCTAAC
TB_IS6110_2_P	SFC620-TGTGGGTAGCAGACCTCACCTATGT-BHQ-2
TB_IS6110_2_R	GTAGGCGTCGGTGACAAA
TB_IS6110_3_F	TCGACCTGAAAGACGTTATCCA
TB_IS6110_3_P	SFC620-CTCGCCGAGGCAGGCATCCA-BHQ-2
TB_IS6110_3_R	CGTTGATCGTCTCGGCTAGTG
TB_ERV3_F	TTTCACACTAACCGCCTCTTC
TB_ERV3_P	HEX-ACCTGCACCATCAAGTCAGAGCAT-BHQ-1

TB_ERV3_R	CCCAGCGAGCAATACAGAAT
TB_rpoB_F	TCGCCGCGATCAAGGAGT
TB_rpoB_P	SFC620-AGCTGAGCCAATTCATGGACCAGA-BHQ-2
TB_rpoB_R	GGGGTTTCGATCGGGCA

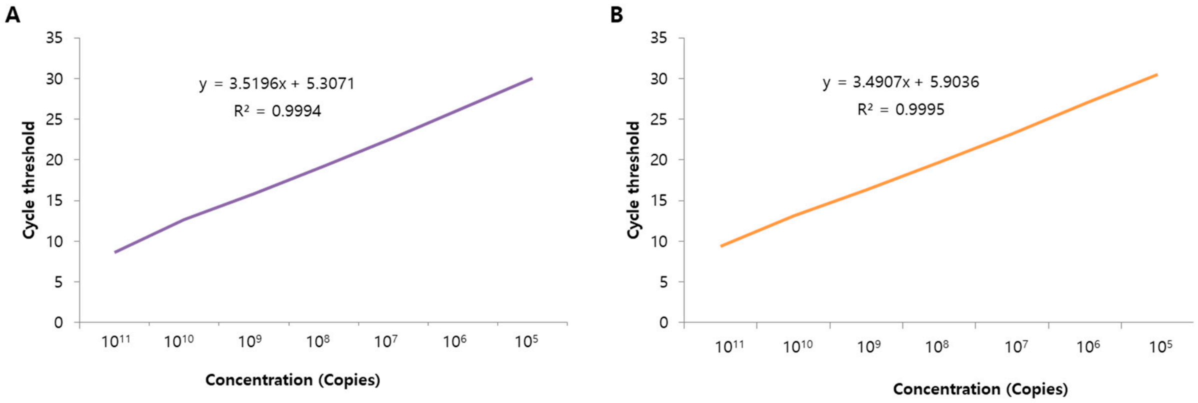

Figure S1. The efficiency of the TB detection kit. The efficiency of the TB detection kit was confirmed using the gene synthesized with the target gene sequence in accordance with the manufacturer's instructions. The standard curve for (A) *IS6110* gene and (B) *ERV3* gene.

Table S2. Results of the TB detection kit for standard curve.

	**Concentration (Copies)**	**Ct Mean**	**Ct SD**
	10^{11}	8.66	0.028284
	10^{10}	12.69	0.070711
	10^{9}	15.875	0.003536
IS6110	10^{8}	19.255	0.003536
	10^{7}	22.73	0.06364
	10^{6}	26.415	0.03182
	10^{5}	30.075	0.081317
	10^{11}	9.33	0.028284
	10^{10}	13.17	0.056569
	10^{9}	16.32	0.007071
ERV3	10^{8}	19.71	0
	10^{7}	23.15	0.028284
	10^{6}	26.915	0.038891
	10^{5}	30.47	0.077782

Table S3. Results of the experiment for 88 clinical samples.

No.	Collected center	Diagnostic results	MTB PCR	Xpert MTB/RIF	SMEAR	CULTURE	This system
1	Severance Hospital	Negative	ND	Negative	Negative	No growth	Negative
2	Severance Hospital	Negative	ND	Negative	Negative	No growth	Negative
3	Severance Hospital	Negative	ND	Negative	Negative	No growth	Positive
4	Severance Hospital	Negative	ND	Negative	Negative	No growth	Positive
5	Severance Hospital	Negative	ND	Negative	Negative	No growth	Positive
6	Severance Hospital	Negative	ND	Negative	Negative	No growth	Negative
7	Severance Hospital	Negative	ND	Negative	Negative	No growth	Negative
8	Severance Hospital	Negative	ND	Negative	Negative	No growth	Positive
9	Severance Hospital	Negative	ND	Negative	Negative	No growth	Positive
10	Severance Hospital	Negative	ND	Negative	Negative	No growth	Negative
11	Severance Hospital	Negative	Negative	ND	Negative	No growth	Negative
12	Severance Hospital	Negative	Negative	Negative	Negative	No growth	Negative
13	Severance Hospital	Negative	ND	Negative	Negative	No growth	Negative
14	Severance Hospital	Negative	ND	Negative	Negative	No growth	Positive
15	Severance Hospital	Negative	ND	Negative	Negative	No growth	Negative
16	Severance Hospital	Negative	ND	Negative	Negative	No growth	Positive
17	Severance Hospital	Negative	ND	Negative	Negative	No growth	Negative
18	Severance Hospital	Negative	ND	Negative	Negative	No growth	Positive
19	Severance Hospital	Negative	ND	Negative	Negative	No growth	Negative
20	Severance Hospital	Negative	ND	Negative	Negative	No growth	Negative
21	Severance Hospital	Negative	ND	Negative	Negative	No growth	Negative
22	Severance Hospital	Negative	ND	Negative	Negative	No growth	Negative
23	Severance Hospital	Negative	ND	Negative	Negative	No growth	Negative
24	Severance Hospital	Negative	ND	Negative	Negative	No growth	Positive
25	Severance Hospital	Negative	ND	Negative	Negative	No growth	Negative
26	Asan Medical Center	Negative	ND	Negative	Negative	No gowth	Negative
27	Asan Medical Center	Negative	Negative	Negative	Negative	No gowth	Negative
28	Asan Medical Center	Negative	Negative	Negative	Negative	No gowth	Negative
29	Asan Medical Center	Negative	Negative	Negative	Negative	No gowth	Positive
30	Asan Medical Center	Negative	Negative	Negative	Negative	No gowth	Negative
31	Asan Medical Center	Negative	ND	Negative	Negative	No gowth	Positive
32	Asan Medical Center	Negative	ND	Negative	Negative	No gowth	Negative
33	Asan Medical Center	Negative	ND	Negative	Negative	No gowth	Negative
34	Asan Medical Center	Negative	ND	Negative	Negative	No gowth	Positive
35	Asan Medical Center	Negative	ND	Negative	Negative	No gowth	Positive
36	Asan Medical Center	Negative	Negative	Negative	Negative	No gowth	Negative
37	Asan Medical Center	Negative	ND	Negative	Negative	No gowth	Negative
38	Asan Medical Center	Negative	Negative	Negative	Negative	No gowth	Negative
39	Asan Medical Center	Negative	ND	Negative	Negative	No gowth	Negative
40	Asan Medical Center	Negative	ND	Negative	Negative	No gowth	Negative
41	Asan Medical Center	Negative	ND	Negative	Negative	No gowth	Negative
42	Asan Medical Center	Negative	Negative	Negative	Negative	No gowth	Positive
43	Asan Medical Center	Negative	ND	Negative	Negative	No gowth	Positive
44	Asan Medical Center	Negative	ND	Negative	Negative	No gowth	Negative
45	Asan Medical Center	Negative	ND	Negative	Negative	No gowth	Negative
46	Asan Medical Center	Negative	Negative	Negative	Negative	No growth	Negative
47	Asan Medical Center	Negative	ND	Negative	Negative	No growth	Negative
48	Asan Medical Center	Negative	ND	Negative	Negative	No growth	Negative
49	Asan Medical Center	Negative	Negative	Negative	Negative	No gowth	Negative

50	Asan Medical Center	Negative	ND	Negative	Negative	No growth	Negative
51	Asan Medical Center	Negative	Negative	Negative	Negative	No growth	Negative
52	Asan Medical Center	Negative	ND	Negative	Negative	No growth	Negative
53	Asan Medical Center	Negative	ND	Negative	Negative	No growth	Negative
54	Asan Medical Center	Negative	ND	Negative	Negative	No growth	Negative
55	Asan Medical Center	Negative	ND	Negative	Negative	No growth	Negative
56	Asan Medical Center	Negative	Negative	Negative	Negative	No gowth	Negative
57	Asan Medical Center	Negative	ND	Negative	Negative	No gowth	Negative
58	Asan Medical Center	Negative	ND	Negative	Negative	No gowth	Negative
59	Asan Medical Center	Negative	Negative	Negative	Negative	No gowth	Negative
60	Severance Hospital	Positive	ND	ND	Negative	MTB	Negative
61	Severance Hospital	Positive	ND	Positive	Negative	MTB	Positive
62	Severance Hospital	Positive	Negative	Negative	Negative	MTB	Negative
63	Severance Hospital	Positive	Negative	Negative	Negative	MTB	Negative
64	Severance Hospital	Positive	ND	Negative	Negative	MTB	Negative
65	Severance Hospital	Positive	ND	Negative	Negative	MTB	Positive
66	Severance Hospital	Positive	ND	Positive	Negative	MTB	Positive
67	Severance Hospital	Positive	ND	Negative	Negative	MTB	Negative
68	Severance Hospital	Positive	ND	Positive	Negative	MTB	Positive
69	Severance Hospital	Positive	Positive	Negative	Negative	MTB	Negative
70	Severance Hospital	Positive	Negative	Negative	Negative	MTB	Positive
71	Severance Hospital	Positive	ND	Negative	Negative	MTB	Positive
72	Severance Hospital	Positive	ND	Negative	Negative	MTB	Positive
73	Severance Hospital	Positive	ND	Negative	Negative	MTB	Positive
74	Asan Medical Center	Positive	ND	Positive	Positive	MTB	Positive
75	Asan Medical Center	Positive	Negative	Negative	Negative	MTB	Positive
76	Asan Medical Center	Positive	ND	Positive	Negative	MTB	Positive
77	Asan Medical Center	Positive	Positive	Positive	Positive	MTB	Positive
78	Asan Medical Center	Positive	ND	Negative	Negative	MTB	Positive
79	Asan Medical Center	Positive	ND	Positive	Negative	MTB	Positive
80	Asan Medical Center	Positive	ND	Positive	Positive	MTB	Positive
81	Asan Medical Center	Positive	ND	Negative	Negative	MTB	Positive
82	Asan Medical Center	Positive	Positive	Positive	Positive	MTB	Positive
83	Asan Medical Center	Positive	ND	Positive	Negative	MTB	Positive
84	Yongin Severance Hospital	Positive	Negative	Positive	Negative	MTB	Positive
85	Yongin Severance Hospital	Positive	Negative	Positive	Negative	MTB	Positive
86	Yongin Severance Hospital	Positive	Negative	Negative	Negative	MTB	Negative
87	Yongin Severance Hospital	Positive	Negative	Positive	Negative	MTB	Negative
88	Dongguk University Ilsan Hospital	Positive	ND	Positive	Negative	MTB	Positive

*ND: not determined

Table S4. Comparison of the new molecular diagnostic system with other TB detection methods.

	**Time**	**Cost per sample ($)**	**LOD***	**Step**	**Special equipment**	**Ref.**
**This study**	3 hr	8.2	9 copies DNA/ul	Sample preparation Detection	Incubater real-time PCR system	-
**Xpert MTB/RIF**	2 hr	9·98 (each cartrige)	131 cfu/mL	Sample preparation Detection	Xpert MTB/RIF	[16]
**MTB PCR**	3 hr	5	10^7 copies H37Rv/ml	Detection	Thermocycler	[31]
**AFB smear**	2 - 8 week	0.26 – 10.50	10000 cfu/mL	Detection	Microscope	[30]
**Mycobacterial Culture**	2 - 8 week	1.63 – 62.01	10 – 50 cfu/mL	Detection	Incubater	[30]

*LOD: limit of detection
